# Dr. Alexander Ennis

**Published:** 1914-04

**Authors:** 


Dr. Alexander Ennis, Albany, 1855, died in Pattersonville, N. Y., February 16, aged 84.

